# Randomized controlled trial evaluating mixed reality technology for training scrub nurses in anterior cervical spine surgery

**DOI:** 10.3389/fmed.2026.1818354

**Published:** 2026-05-13

**Authors:** Wenqing Yang, Tingting Ma

**Affiliations:** Department of Operating Room, The Affiliated Suqian Hospital of Xuzhou Medical University, Suqian, China

**Keywords:** cognitive load, mixed reality, operating room, randomized controlled trial, spinal surgery, surgical nursing education

## Abstract

**Background:**

Mastery of instrument sequencing and spatial anatomy is crucial for scrub nurses in anterior cervical spine surgery (ACSS), yet conventional training often fails to provide immersive, three-dimensional practice.

**Aim:**

This randomized controlled trial evaluated a novel Mixed Reality (MR) training module against standard training for ACSS scrub nurse education.

**Methods:**

Sixty nurses were randomly assigned to an MR training group (*n* = 30) or a traditional training group (*n* = 30). The intervention group completed a structured MR curriculum using Microsoft HoloLens 2, featuring interactive 3D anatomy, virtual instrument handling, and emergency scenario simulation. The control group received standard lectures and video-based instruction. Primary outcomes were scores on theoretical knowledge, practical performance, and emergency response assessments. Secondary outcomes included cognitive load (NASA-TLX) and training satisfaction.

**Results:**

The MR group achieved significantly higher post-training scores in theoretical knowledge (92.47 vs. 86.33, *p* < 0.001), practical performance (94.50 vs. 88.97, *p* < 0.001), and emergency response (91.73 vs. 85.40, *p* < 0.001). Participants in the MR group also reported a significantly lower overall cognitive load (NASA-TLX total: 28.60 vs. 36.43, *p* < 0.001) and markedly higher satisfaction across all measured domains (*p* < 0.001).

**Conclusion:**

Immersive MR training is more effective than traditional methods in developing essential knowledge, skills, and situational preparedness for ACSS. It facilitates learning by reducing cognitive burden and increasing engagement, presenting a valuable tool for advancing surgical nursing education.

## 1 Introduction

Anterior cervical spine surgery (ACSS) is a definitive treatment for cervical disc herniation and spinal stenosis (1). The procedure involves a complex surgical field adjacent to critical structures including the trachea, esophagus, carotid artery, and spinal cord. This anatomical complexity demands that scrub nurses possess an exceptional understanding of spatial relationships, precise instrument knowledge, and the ability to anticipate surgical steps and manage intraoperative contingencies (2, 3).

Conventional training for scrub nurses typically incorporates didactic lectures, two-dimensional anatomical images, surgical video observations, and supervised operating room apprenticeships (4). While foundational, these methods present notable constraints. Two-dimensional materials fail to convey intricate three-dimensional anatomy and surgical approaches effectively. Video observation is a passive learning activity with limited engagement. Furthermore, hands-on apprenticeship opportunities are constrained by operating room schedules, patient safety considerations, and the inherent high-stakes environment, which limits repetitive practice and error management in a risk-free setting.

Extended Reality (XR) technologies, particularly Mixed Reality (MR), are emerging as transformative tools in medical education (2). MR seamlessly integrates interactive digital content into the user’s real-world environment, creating immersive, spatially aware learning experiences. In surgical training for physicians, MR has demonstrated efficacy in enhancing anatomical understanding and procedural planning for complex structures like the pelvis and skull base (5). However, its application and systematic evaluation in the education of operating room nursing staff, specifically for technically demanding subspecialties like spine surgery, remain largely unexplored.

Cognitive Load Theory (CLT) provides a useful framework for understanding the potential advantages of MR-based training. CLT posits that learning is hindered when extraneous cognitive load—caused by inefficient instructional design—consumes limited working memory resources (6). Traditional training methods, which require learners to mentally reconstruct three-dimensional anatomy from two-dimensional images, impose a high extraneous load. By presenting information in a spatially coherent, three-dimensional format, MR may reduce this cognitive burden, freeing resources for schema acquisition and skill development (7).

Recent studies have begun to explore the application of MR technologies such as Microsoft HoloLens 2 in surgical nursing education. For example, some studies have demonstrated the feasibility of MR for instrument recognition training in orthopedic settings (5), and positive outcomes for situational awareness training using head-mounted displays have also been reported (8). However, to our knowledge, no randomized controlled trial has systematically evaluated MR-based training specifically for scrub nurses in anterior cervical spine surgery.

This study addresses this gap by developing and implementing a targeted MR training module for ACSS scrub nurse education. We conducted a randomized controlled trial to rigorously compare the efficacy of this innovative approach against established traditional training methods. We hypothesized that MR-based training would lead to superior learning outcomes, reduced cognitive load during skill acquisition, and higher learner satisfaction (9).

## 2 Methods

### 2.1 Study design and participants

From June to December 2023, a parallel-group, randomized controlled trial was conducted within the operating room department of our institution. The study protocol received approval from the Ethics Review Committee of Suqian Hospital Affiliated to Xuzhou Medical University (Approval No: IRB-S-2023012). Written informed consent was obtained from all participants prior to their enrollment.

Eligible participants were registered nurses with at least 1 year of general operating room experience but no prior independent scrub nurse role in ACSS. Nurses with a history of vestibular disorders, epilepsy, or who had participated in similar training within the preceding 3 months were excluded.

*A priori* sample size calculation was performed using G*Power 3.1 (10). Based on a pilot study indicating a large expected effect size (*d* = 0.8) for the primary outcome (practical performance score), with *α* = 0.05 and power (1 − *β*) = 0.90, a minimum of 26 participants per group was required. Accounting for a potential 15% attrition rate, 60 nurses were recruited.

### 2.2 Randomization and blinding

The 60 enrolled participants were randomly allocated in a 1:1 ratio to either the MR training group or the Traditional training group. Allocation was performed by an independent statistician using a computer-generated random number sequence, concealed in sequentially numbered, opaque envelopes. Due to the nature of the intervention, participants and trainers could not be blinded to group assignment. However, outcome assessors for the practical and emergency response assessments were blinded to group allocation.

### 2.3 Transparency and protocol disclosure

This study was designed as an educational intervention trial without patient-related outcomes. At the time of study initiation, prospective registration was not mandated by institutional policy for non-clinical educational studies. To ensure transparency, the full study protocol, raw data, and ethical documentation are available from the corresponding author upon reasonable request.

### 2.4 Interventions

#### 2.4.1 Traditional training group (control)

Participants received standard training over 4 h, consisting of:

A 2-h lecture from a senior spine surgery nurse educator and an orthopedic surgeon, covering ACSS anatomy, procedural steps, instrument names/functions, and key nursing considerations.
A 1-h session observing two standardized ACSS procedure videos.
A 1-h hands-on session for physical identification and handling of the actual surgical instrument sets on a sterile table.

#### 2.4.2 MR training group (intervention)

This group received the same 2-h foundational lecture as the control group. Subsequently, they undertook a 2-h immersive training session using a custom-developed “ACSS MR Training Module” deployed on the Microsoft HoloLens 2 platform.

The training module was developed in collaboration with the institutional simulation center. Three-dimensional anatomical models were reconstructed from de-identified CT and MRI data of a representative patient to ensure anatomical accuracy of key structures, including the cervical vertebrae, intervertebral discs, trachea, esophagus, and carotid arteries. Instrument models were created based on high-resolution digital photographs of the actual surgical instruments used in our institution. The module was designed to support interactive hand gestures and voice commands, allowing participants to manipulate virtual objects in a spatially anchored environment.

The module comprised the following components:

*3D anatomical exploration*: a dynamic, multi-layered 3D model of the cervical spine (from skin to bone) allowing 360° rotation and stepwise dissection to visualize the standard anterior (Smith-Robinson) approach.
*Interactive instrument training*: high-fidelity 3D models of all critical ACSS instruments (e.g., Caspar retractors, cervical drills, curettes, anterior plating systems). Using hand gestures and voice commands, participants practiced selecting and “virtually passing” the correct instrument in response to audio prompts of surgical steps. Immediate corrective feedback was provided for errors.
*Emergency scenario simulation*: interactive modules simulating intraoperative challenges (e.g., screw stripping, graft dislodgement, increased bleeding), requiring the participant to identify the issue and select the appropriate corrective instrument or action.

### 2.5 Outcome measures

Assessments were conducted 1 week after training completion by blinded assessors.

#### 2.5.1 Theoretical knowledge

A 100-point multiple-choice and short-answer test covering anatomy, instrumentation, procedural sequence, and complication management. The test was developed by two senior spine surgeons and one senior nursing educator, reviewed by an external panel of three surgical education experts, and pilot-tested with 10 operating room nurses (not included in the study) to establish face validity and appropriate difficulty.

#### 2.5.2 Practical performance

In a simulated OR setting, participants performed as the scrub nurse for a standardized ACSS procedure on a spine model. Performance was rated by two blinded expert nurses using a 10-item checklist scoring instrument preparation, passing accuracy, timeliness, and aseptic technique (0–100). The checklist was developed based on a review of established scrub nurse competency frameworks [citations] and refined through iterative discussion with three senior spine surgery nurses (each with >10 years of experience) to ensure content relevance. During pilot testing with 10 operating room nurses not included in the main study, inter-rater reliability between the two assessors was excellent (intraclass correlation coefficient = 0.89), and internal consistency was acceptable (Cronbach’s *α* = 0.89).

#### 2.5.3 Comprehensive emergency response

Response was scored on a 5-item scale (0–100) assessing problem identification, communication, and solution correctness. This scale was adapted from the validated Non-Technical Skills for Surgeons (NOTSS) framework and modified for scrub nurse roles.

#### 2.5.4 Cognitive load

Measured immediately post-training using the NASA Task Load Index (NASA-TLX), which rates mental, physical, and temporal demand, effort, performance, and frustration on 0–100 scales. A weighted overall score (0–100) was calculated.

#### 2.5.5 Training satisfaction

Assessed via a 10-item, 5-point Likert scale questionnaire covering interest, comprehensibility, perceived skill gain, method appeal, and overall satisfaction (total score 10–50). The questionnaire was constructed based on the Simulation Effectiveness Tool-Modified (SET-M) and adapted for the MR context. Confirmatory factor analysis confirmed a single-factor structure explaining 68.4% of the variance, and internal consistency was high (Cronbach’s *α* = 0.92).

### 2.6 Statistical analysis

Data were analyzed using SPSS 26.0. Normality was confirmed using Shapiro–Wilk tests. Between-group comparisons of continuous outcomes were made using independent samples t-tests. Categorical baseline data were compared using Chi-square tests. A two-tailed *p*-value <0.05 was considered statistically significant. Data are presented as mean ± standard deviation (SD).

## 3 Results

All 60 participants completed the study protocol with no dropouts. No adverse effects, including cybersickness, nausea, dizziness, or visual discomfort, were reported by participants in the MR group during or immediately after the training session. Participants were screened for vestibular disorders prior to enrollment (see exclusion criteria), which may have contributed to the absence of these events. Baseline demographic and professional characteristics were well-balanced between the two groups (Table 1), confirming successful randomization.

**Table 1:** Baseline characteristics of participants.

Characteristic	Experimental group (*n* = 30)	Control group (*n* = 30)	Statistics	*p*-value
Age (years, mean ± SD)	28.43 ± 3.67	29.10 ± 4.12	*t* = −0.67	0.505
Gender [*n* (%)]			*χ*^2^ = 0.14	0.708
Male	4 (13.3)	3 (10.0)		
Female	26 (86.7)	27 (90.0)		
Education level [*n* (%)]			*χ*^2^ = 0.27	0.874
Associate degree	5 (16.7)	4 (13.3)		
Bachelor’s degree	23 (76.7)	24 (80.0)		
Master’s degree	2 (6.6)	2 (6.7)		
OR experience (years, Mean ± SD)	5.37 ± 2.45	5.83 ± 2.89	*t* = −0.68	0.501

OR, Operating Room; SD, Standard Deviation. Independent samples *t*-test was used for continuous variables; Chi-square test was used for categorical variables.

### 3.1 Primary outcomes: knowledge and skills assessment

The MR training group demonstrated significantly superior performance across all assessed domains compared to the traditional training group (Table 2).

**Table 2:** Post-training assessment scores (mean ± SD).

Assessment domain (score range)	MR group (*n* = 30)	Traditional group (*n* = 30)	Mean difference (95% CI)	*t*-value	*p*-value	Cohen’s d
Theoretical knowledge (0–100)	92.47 ± 3.21	86.33 ± 4.15	6.14 (4.32, 7.96)	6.42	<0.001	**1.31**
Practical performance (0–100)	94.50 ± 2.88	88.97 ± 3.76	5.53 (3.75, 7.31)	6.62	<0.001	**1.29**
Emergency response (0–100)	91.73 ± 3.05	85.40 ± 4.22	6.33 (4.44, 8.22)	6.77	<0.001	**1.32**

Independent samples *t*-test was used for comparisons. Cohen’s d values ≥0.8 indicate large effect sizes. Bold values are Cohen’s d effect sizes.

### 3.2 Secondary outcomes: cognitive load and satisfaction

Participants in the MR group reported a significantly lower overall cognitive load (NASA-TLX total score) and lower scores on the mental, physical, temporal, effort, and frustration subscales. Conversely, their self-rated performance was significantly higher (Table 3). Satisfaction with the training was markedly greater in the MR group across all measured dimensions (Table 4).

**Table 3:** Comparison of NASA-TLX cognitive load ratings between the two groups (mean ± SD).

NASA-TLX dimension	MR group (*n* = 30)	Traditional group (*n* = 30)	Mean difference (95% CI)	*t*-value	*p*-value	Cohen’s d
Mental demand	45.33 ± 6.52	58.67 ± 8.91	−13.34 (−17.21, −9.47)	−6.89	<0.001	**−1.68**
Physical demand	30.20 ± 5.44	38.50 ± 7.23	−8.30 (−11.50, −5.10)	−5.12	<0.001	**−1.30**
Temporal demand	32.77 ± 6.18	49.30 ± 8.45	−16.53 (−20.32, −12.74)	−8.85	<0.001	**−2.20**
Effort	48.83 ± 7.02	62.17 ± 9.36	−13.34 (−17.52, −9.16)	−6.35	<0.001	**−1.60**
Performance	79.50 ± 8.11	68.43 ± 9.87	11.07 (6.50, 15.64)	4.88	<0.001	**1.22**
Frustration	25.17 ± 5.89	41.50 ± 8.74	−16.33 (−20.12, −12.54)	−8.74	<0.001	**−2.17**
Total score	28.60 ± 4.33	36.43 ± 5.21	−7.83 (−10.22, −5.44)	−6.71	<0.001	**−1.63**

SD, Standard Deviation; NASA-TLX, National Aeronautics and Space Administration Task Load Index. Scores range from 0 to 100 for each dimension. A higher score on the Performance dimension indicates better self-perceived performance, whereas a higher score on other dimensions and the total score indicates greater cognitive load. Independent samples t-test was used for comparisons. Negative Cohen’s d values indicate lower cognitive load in the MR group (favorable outcome); positive d values for the Performance dimension indicate higher self-rated performance in the MR group. Cohen’s d values ≥0.8 (absolute value) indicate large effect sizes. Bold values are Cohen’s d effect sizes.

**Table 4:** Comparison of training satisfaction scores between the two groups (mean ± SD).

Satisfaction dimension	MR group (*n* = 30)	Traditional group (*n* = 30)	Mean difference (95% CI)	*t*-value	*p*-value	Cohen’s d
Stimulation of interest	4.63 ± 0.49	3.80 ± 0.61	0.83 (0.55, 1.11)	6.06	<0.001	**1.49**
Content comprehensibility	4.57 ± 0.50	3.93 ± 0.58	0.64 (0.36, 0.92)	4.71	<0.001	**1.18**
Perceived skill improvement	4.70 ± 0.47	4.03 ± 0.67	0.67 (0.37, 0.97)	4.59	<0.001	**1.16**
Attractiveness of training method	4.80 ± 0.41	3.60 ± 0.77	1.20 (0.89, 1.51)	7.82	<0.001	**1.91**
Overall satisfaction	4.67 ± 0.48	3.97 ± 0.67	0.70 (0.40, 1.00)	4.81	<0.001	**1.20**
Total satisfaction score	46.73 ± 2.08	38.67 ± 3.12	8.06 (6.69, 9.43)	11.97	<0.001	**3.00**

SD, Standard Deviation. Each dimension was rated on a 5-point Likert scale (1 = Strongly Disagree to 5 = Strongly Agree). The total score ranges from 10 to 50. Independent samples *t*-test was used for comparisons. Cohen’s d values ≥0.8 indicate large effect sizes. Bold values are Cohen’s d effect sizes.

## 4 Discussion

### 4.1 Cognitive offloading and procedural embodiment may explain enhanced learning

This study demonstrates that MR-based training significantly outperforms traditional methods across all primary learning outcomes for scrub nurses in ACSS. While our randomized controlled trial provides clear evidence of superior effectiveness, the underlying cognitive mechanisms remain inferential. The following discussion offers theoretically grounded interpretations that should be tested in future mechanistic studies.

The observed superiority may be partly explained by MR’s ability to restructure the cognitive architecture of learning. The significantly lower scores for mental and temporal demand in the MR group (Table 3) suggest a reduction in extraneous cognitive load, consistent with Cognitive Load Theory. Traditional methods require learners to perform an inefficient “spatial translation”—constructing 3D mental models from 2D resources—which heavily burdens working memory (11). MR appears to reduce the need for this step by providing an immediately accessible, spatially coherent 3D representation of anatomy and instruments. This potential for cognitive “offloading” may allow freed mental resources to be directed toward schema acquisition and automation of procedural sequences. Consequently, the repetitive “virtual passing” in MR could function as a form of procedural embodiment, where gesture-based interaction helps build sensorimotor schemas that may be transferable to the physical world. This interpretation is consistent with the concurrent improvements observed in both declarative knowledge (12) (theoretical scores) and applied psychomotor skill (practical performance).

### 4.2 Fostering situational awareness and team preparedness: the clinical translation

The potential clinical value of this training paradigm may extend beyond individual competency metrics to possible enhancements in surgical team performance and patient safety. The MR group’s superior performance in the comprehensive emergency response assessment is particularly telling (91.73 vs. 85.40, *p* < 0.001). In complex surgeries like ACSS, a scrub nurse’s critical role involves not only routine tasks but also managing unexpected events (13). MR simulation provides a safe, repeatable platform for the cognitive rehearsal of crises—an opportunity that is difficult to achieve through passive observation alone. This type of practice may contribute to developing situational awareness: the perception of key elements (14), comprehension of their meaning, and projection of future status within a dynamic environment (15, 16). A nurse who has virtually “experienced” a complication might recognize its precursors earlier and respond more effectively (17). Furthermore, by visualizing the surgical field from the surgeon’s perspective and understanding anatomical constraints in 3D, MR training may have the potential to foster a stronger shared mental model within the OR team (18). Such shared understanding is widely believed to support fluent communication, anticipatory support, and ultimately safer and more efficient surgery, although these downstream effects remain to be directly tested in clinical settings.

### 4.3 Limitations and future directions

While this study presents compelling evidence for the efficacy of MR training, several limitations must be acknowledged.

First, the transfer of skills from simulation to live operating rooms remains to be formally evaluated. Second, the single-center design and modest sample size (*n* = 60) limit generalizability; multi-center replication is needed. Third, the novelty of the MR intervention may have introduced a Hawthorne effect, whereby participants’ performance may have been enhanced by awareness of being part of an innovative pilot rather than solely by the educational content. Future studies should consider a sham technology control group (e.g., using HoloLens 2 to view 2D videos) to isolate the specific effect of MR content. Fourth, the high initial investment for MR hardware and software development warrants formal cost-effectiveness analyses to determine whether the observed improvements justify widespread adoption. Fifth, we did not assess long-term skill retention beyond 1 week; it remains unclear whether the observed advantages persist at 3, 6, or 12 months. Longitudinal follow-up is essential to evaluate the durability of training effects and to determine the appropriate frequency of refresher training. Finally, while participants were screened for vestibular disorders, future studies should include standardized assessments of cybersickness.

Addressing these limitations through multi-center trials, longitudinal designs, and cost-effectiveness analyses will be critical to define the role of MR training in surgical nursing education (19).

### 4.4 Economic considerations

The initial investment for MR hardware (e.g., Microsoft HoloLens 2) and custom software development presents a practical barrier to widespread adoption. In this study, the MR group outperformed the traditional group by approximately 5–6 points across primary outcomes. Whether this magnitude of improvement justifies the upfront cost depends on institutional context, training volume, and long-term outcomes. Traditional training incurs ongoing costs for educator time, facility use, and consumables, whereas MR training involves higher initial costs but may offer economies of scale for repeated use once the module is developed. Formal cost-effectiveness analyses—comparing MR against both traditional training and lower-cost alternatives such as video-based simulation—are warranted before recommending widespread implementation. Such analyses should consider not only direct costs but also potential indirect benefits, including reduced operating room time, improved team efficiency, and enhanced patient safety outcomes.

## 5 Conclusion

In this controlled simulation-based setting, immersive MR training demonstrated superior short-term outcomes compared to traditional methods for developing essential knowledge, skills, and situational preparedness for ACSS. The intervention facilitated learning by reducing cognitive burden and increasing engagement, providing a cognitively efficient approach that offloads spatial translation burdens and enables embodied skill practice (17). However, further research—including multi-center trials, assessments of long-term skill retention, and cost-effectiveness analyses—is essential before MR training can be recommended for widespread integration into standard surgical nursing education.

## Data Availability

The raw data supporting the conclusions of this article will be made available by the authors, without undue reservation.
